# Assessment of pain levels in patients undergoing periodontal versus endodontic treatment: A cross-sectional observational study

**DOI:** 10.6026/973206300211779

**Published:** 2025-06-30

**Authors:** Ashutosh Kumar, Saleem Azhar, Swati Rai, Mansha Zarabi, Om Suman Bharti, Kafilul Bari Khan

**Affiliations:** 1 Department of Conservative Dentistry and Endodontics, Hitkarini Dental College and Hospital, Jabalpur, Madhya Pradesh, India; 2 Department of Conservative Dentistry & Endodontics, Teerthanker Mahaveer Dental College & Research Centre, Moradabad, Uttar Pradesh, India; 3 Department of Pharmacology, Autonomous State Medical College Sultanpur, Uttar Pradesh, India; 4 Department of Periodontics and Oral Implantology, Desh Bhagat Dental College and Hospital, Mandi Gobindgarh, Punjab, India; 5 Department of Periodontology, Mithila Minority Dental College and Hospital, Darbhanga, Bihar, India; 6 Department of Conservative Dentistry and Endodontics, Kalinga Institute of Dental Sciences, Bhubaneswar, Odisha, India

**Keywords:** Endodontic treatment, pain assessment, pain management, periodontal treatment, post-operative pain

## Abstract

Pain levels in patients undergoing periodontal and endodontic treatment is of interest. Using pain assessment tools like the Visual Analog Scale (VAS), Numerical Rating Scale (NRS), and McGill Pain Questionnaire (MPQ), we assessed pain before, during, and after treatment. Results indicated that endodontic treatment caused higher pain levels, especially post-operatively, compared to periodontal treatment. The findings highlight the need for tailored pain management strategies in endodontics. These insights can guide clinicians in improving pain management for patients undergoing dental procedures.

## Background:

Many dental and medical treatments include discomfort as an unavoidable side effect. In dentistry, pain management controls basically patient comfort and compliance [1]. Among the several kinds of dental operations, two often utilized ones, each of which can be associated with different degrees of pain, are periodontal and endodontic treatments. Although a direct comparison of pain levels felt by patients receiving periodontal rather than endodontic procedures is rare, in practical terms, it is fairly important [2]. The different scopes, techniques, and ultimate goals of periodontal and endodontic treatments can affect the type and degree of discomfort patients' experience. Targeting largely the prevention, diagnosis, and treatment of periodontal illnesses, periodontal treatment mostly influences the supporting elements of the teeth, such as the gums and bone. Including gum surgery, scaling and root planing, and other therapies aimed at reducing inflammation and infection, it also involves procedures often referred to as root canal treatment, also known as endodontic treatment, which treats pulp (soft tissue inside the tooth) illnesses or diseases, as well as surrounding tissues [3]. Typically involving the removal of diseased tissue, cleaning, shaping, and sealing of the root canal system, endodontics primarily seeks to repair a tooth that has been severely damaged by trauma or infection. While both treatments are necessary for maintaining dental health, the type of operations performed and the anatomical sites involved can cause varying degrees of pain [4]. For example, whereas endodontic treatments target the pulp, which is innervated and can cause significant discomfort, periodontal therapies often come into contact with very sensitive soft tissues, such as the gums. Knowing the degree of pain connected with every operation would thus enable doctors to create more effective pain control plans [5]. While studies comparing pain between periodontal and endodontic treatments are few, the corpus of research on pain related to dental treatments is expanding. While some studies indicate that periodontal treatments are more painful due to the involvement of sensitive periodontal tissues, others assert that root canal treatments are connected with significant pain, especially post-operatively, due to inflammation and irritation of the pulp and surrounding structures [6]. Still, a number of elements can affect the pain experience, including patient anxiety, personal pain tolerance, the degree of the disease being treated, and the kind of anaesthesia used [7]. Dental pain management has undergone recent changes in response to novel anaesthetic approaches, improved pharmacological treatments, and a deeper understanding of pain processes. Even if sedative methods-including conscious sedation and nitrous oxide-have also helped to minimize discomfort, local anaesthesia is still the pillar of pain control during dental procedures. Usually, post-operative pain management is accomplished using analgesics; NSAIDs and opioids aid with discomfort [8]. Although doctors should counsel patients carefully, the availability of over-the-counter medications has also made it simpler for patients to self-manage their post-operative discomfort [9]. Notwithstanding these advances, appropriate therapy and improvement of patient outcomes depend on an awareness of the pain dynamics both during and after periodontal and endodontic operations. Many pain measuring scales have been applied in dentistry research: the Numerical Rating Scale (NRS), the Visual Analogue Scale (VAS), and the McGill Pain Questionnaire (MPQ). These tools assess the degree of pain and its impact on a patient's quality of life, thereby providing precise statistics for analysis and comparison. This current effort aims to provide a comprehensive, evidence-based comparison by evaluating the discomfort experienced by people undergoing two conventional dental treatments using consistent methodologies [10]. Many factors can affect how one experiences pain during dental procedures; therefore, significant focus should be placed on these factors when assessing pain intensity. For example, pain signals can become more intense during fear and discomfort. Thus, in addition to medical treatment, suitable psychological support is rather important. Furthermore, factors affecting the degree of pain experienced include the type and dosage of anaesthesia administered the dental professional's knowledge, and the complexity of the treatment. Knowing these links helps one design appropriate painkillers tailored to the patient's needs. Therefore, it is of interest to describe the variations in pain perception between periodontal and endodontic treatments, with the goal of enhancing patient experience and providing valuable insights for improving pain management strategies in both treatments.

## Methodology:

This study's approach focused on comparing pain levels associated with endodontic and periodontal therapy for patients. Designed as a prospective, observational research project with a quantitative approach, the study collected objective data on pain intensity before and after treatment using standardized pain assessment tools. Designed to assess pain levels in patients receiving two distinct dental treatments-periodontal and endodontic procedures-this cross-sectional, observational study followed patients after the operations to evaluate both post-operative and immediate pain. The study gathered information from a diverse population undergoing either periodontal or endodontic treatment in a dental clinic or hospital environment, thereby providing a real-world context for understanding pain experiences.

## Inclusion criteria:

[1] Adults aged 18 to 65 years.

[2] Patients scheduled for either periodontal or endodontic treatment.

[3] Willingness to provide informed consent to participate in the study.

[4] Absence of any severe medical conditions that may interfere with pain perception or treatment (*e.g.*, chronic pain disorders, severe allergies to anaesthetics).

[5] No history of substance abuse or psychological conditions that may affect the pain response.

## Exclusion criteria:

[1] Pregnant or breastfeeding women.

[2] Patients with mental or cognitive impairments that might hinder their ability to understand the pain assessment process.

[3] Patients with a history of significant complications following previous periodontal or endodontic procedures.

[4] Patients currently using systemic corticosteroids, nonsteroidal anti-inflammatory drugs (NSAIDs), or opioids that might interfere with pain perception or the ability to report pain accurately.

The sample size was determined based on a power analysis to achieve statistical significance. A total of 60 participants (30 for each treatment group) were included to ensure a meaningful comparison of pain levels between the two groups. Periodontal treatment involved either a non-surgical procedure (scaling and root planing) or a surgical procedure (*e.g.*, flap surgery or gingivectomy), depending on the patient's clinical needs. Local anaesthesia (Lidocaine 2% with epinephrine) was administered to ensure that patients experienced minimal pain during the procedure. The dentist performed the treatment in accordance with standard clinical guidelines. Following the procedure, patients received post-operative instructions on pain management, including the use of over-the-counter analgesics such as ibuprofen. Endodontic treatment (root canal therapy) involves the cleaning and shaping of the root canal system, followed by filling and sealing. Local anaesthesia (Lidocaine 2% with epinephrine) was used to numb the area around the affected tooth. After the procedure, patients received post-operative instructions on pain management, similar to the periodontal treatment group. This included taking analgesics to manage discomfort. To objectively assess pain levels, several validated pain measurement tools were utilized throughout the study. These tools are reliable and widely used in clinical practice to quantify pain intensity, providing a clear understanding of the patient's pain experience.

## The Visual Analog Scale (VAS) was used to measure pain intensity at multiple points:

## Pre-treatment pain:

Assess baseline discomfort before the procedure.

## During treatment:

Immediate pain levels experienced during the procedure are recorded at specific intervals.

## Post-treatment pain:

Pain levels were assessed immediately after the treatment and at regular intervals (*e.g.*, 6, 12, 24, and 48 hours post-procedure).

The VAS consists of a 10 cm line with two endpoints, "no pain" (0) and "worst possible pain" (10). Patients were asked to mark the point on the line that represented their pain intensity. The score was measured by calculating the distance from the "no pain" end to the patient's mark. In addition to the VAS, the Numerical Rating Scale (NRS) was used to measure pain intensity. This scale is a simple 0-10 scale where 0 represents no pain and 10 describe the worst pain imaginable. Patients were asked to rate their pain at specified times during and after the procedure. The NRS facilitated easy communication of pain levels and proved highly effective in clinical settings. The McGill Pain Questionnaire (MPQ) was administered post-treatment to assess the qualitative aspects of the pain experience, including its nature, intensity, and duration. The MPQ included descriptors such as "sharp," "dull," "throbbing," or "burning," providing a comprehensive overview of the pain experience that extends beyond its intensity.

## Data were collected at three key points during the study:

## Pre-treatment assessment:

Patients were asked to report their baseline pain levels using the Visual Analog Scale (VAS) and Numerical Rating Scale (NRS) before the procedure began. This helped assess any pain due to the underlying dental condition before the intervention. During treatment, pain levels were assessed at regular intervals. Patients were asked to rate their pain on the VAS and NRS after key stages of the treatment (*e.g.*, after local anaesthesia injection, after significant dental manipulation such as scaling or root canal shaping).

## Post-treatment assessment:

Pain was measured six, twelve, twenty-four, forty-eight hours after treatment, as well as soon after the procedure. This also revealed any delayed onset of pain or discomfort, as well as the acute suffering that occurred immediately after the treatment. Furthermore, observation was conducted to assess post-treatment analgesic use (type and dosage), evaluating the efficacy of the advised pain management strategy. Analysis of the acquired data made use of descriptive and inferential statistical methods. Descriptive statistics (mean, median, standard deviation) allowed us to arrange pain levels for both treatment groups: periodontal and endodontic. Using inferential statistics, such as independent t-tests or Mann-Whitney U tests, pain levels between the two groups were compared at each time point. Considered statistically significant was a p-value less than 0.05. Under study were pre-treatment pain levels, age, gender, and whether discomfort was felt before and after the surgery. The study ruled out any potential confusing elements, including operation complexity or anxiety levels. This study aimed to provide a comprehensive comparison of pain levels between periodontal and endodontic treatments; however, it was not without its limitations. Personal pain experience is influenced by psychological factors, including anxiety and fear, which in this observational study were not completely under control. Furthermore, variations in operator skill and patient compliance with post-operative therapy would have influenced pain results. Still, the study's comprehensive pain assessment approach offers significant new insights into the suffering people experience during regular dental procedures.

## Results:

A total of 60 participants were included in the study, with 30 patients undergoing periodontal treatment and 30 patients receiving endodontic treatment. Pain intensity was assessed using the Visual Analog Scale (VAS), Numerical Rating Scale (NRS), and McGill Pain Questionnaire (MPQ). The data collected were analysed using descriptive and inferential statistics to identify significant differences in pain levels between the two treatment groups. Before the start of the procedures, baseline pain levels were measured for both groups. The VAS and NRS scores were recorded to assess the pain associated with the underlying dental conditions (*e.g.*, periodontal disease or pulp infection) prior to treatment (Table 1 - see PDF). Pain levels were assessed at multiple stages during the treatment procedure. The VAS and NRS scores were taken immediately after local anaesthesia injection, during the treatment procedure, and at the end of the treatment (Table 2 - see PDF). During the procedures, both groups experienced minimal pain during anaesthesia administration. However, pain levels rose during the active treatment phase. Periodontal patients generally reported moderate pain (2.5 ± 1.1) during scaling and root planing, while endodontic patients reported slightly higher pain levels (3.2 ± 1.2) during root canal shaping. Post-treatment pain was assessed at 6, 12, 24, and 48 hours after the procedures. The VAS and NRS scores indicated that both groups experienced a moderate level of pain, with a peak in discomfort typically occurring within the first 12 hours (Table 3 - see PDF). Post-treatment pain was significantly higher in the endodontic group, particularly in the first 12 hours following the procedure. This suggests that endodontic treatment, while manageable with pain relief medications, caused more prolonged discomfort compared to periodontal therapy. The McGill Pain Questionnaire (MPQ) was administered 48 hours after the treatment to evaluate the qualitative nature of the pain. Patients in both groups reported a range of pain descriptors, such as "throbbing," "sharp," and "aching." Endodontic patients were more likely to describe their pain as "throbbing" and "dull," while periodontal patients reported pain as more "sharp" and "stinging" (Table 4 - see PDF). Overall, when comparing pain levels across the treatment groups, significant differences were observed. Endodontic treatment generally resulted in higher pain levels during and after the procedure compared to periodontal therapy, especially during the post-operative period. The peak pain intensity for endodontic patients occurred within the first 12 hours, whereas periodontal patients had relatively lower post-treatment pain levels. Independent t-tests revealed that the difference in post-treatment pain levels (as measured by VAS at 6, 12, 24, and 48 hours) was statistically significant between the two treatment groups (p < 0.05), with endodontic patients experiencing more discomfort than periodontal patients. Both groups were provided with similar post-treatment pain management strategies, including nonsteroidal anti-inflammatory drugs (NSAIDs) like ibuprofen. The average dosage of analgesics taken by periodontal patients was 1.5 tablets over the first 48 hours, while endodontic patients required 2.3 tablets on average during the same period. This further indicates that endodontic treatment led to higher pain levels, necessitating greater analgesic use.

## Discussion:

This study assessed and compared pain levels associated with periodontal and endodontic treatments using validated pain assessment tools, including the Visual Analogue Scale (VAS), the Numerical Rating Scale (NRS), and the McGill Pain Questionnaire (MPQ). Our findings indicate that patients undergoing endodontic treatment reported significantly higher pain levels during and after the procedure compared to those receiving periodontal therapy. These results align with and contribute to the growing body of research on dental pain perception. The pre-treatment pain scores in the endodontic group were notably higher (VAS: 4.0 ± 1.7) than those in the periodontal group (VAS: 3.2 ± 1.5), supporting earlier findings by Canakçi *et al.* [8], who reported that pulp-related pathologies typically result in more severe pre-operative discomfort than periodontal conditions. The greater intensity of post-operative pain in endodontic patients, especially within the first 12 hours, further confirms the results of prior studies suggesting that root canal procedures can trigger lingering inflammation and post-treatment sensitivity due to manipulation of the innervated pulp tissue and periapical structures. In contrast, while periodontal procedures, particularly surgical ones, can cause discomfort due to the manipulation of soft tissues, such as the gingiva, they are often better tolerated, possibly because of the absence of direct nerve involvement within the treated regions. This observation is consistent with the work of Sirintawat *et al.* [9], who noted that periodontal pain is often described as sharp but transient and is usually well-managed with local anaesthesia and mild analgesics. The qualitative analysis using the MPQ revealed that endodontic pain was more often described as "throbbing" and "dull," indicating deeper, more persistent discomfort likely due to inflammation within the pulp and periapical tissues. In comparison, periodontal pain descriptors such as "sharp" and "stinging" suggest localised surface irritation, which tends to subside more rapidly. These distinctions align with earlier findings by Maggirias and Locker [10], who highlighted how psychological perceptions and the nature of the underlying pathology influence the characterisation of dental pain. The higher average analgesic consumption in the endodontic group (2.3 tablets over 48 hours) compared to the periodontal group (1.5 tablets) reinforces the quantitative findings and further underscores the need for more robust post-operative pain management strategies in endodontic cases. This outcome supports the recommendations by Becker *et al.* [1], who emphasised the importance of tailored analgesic regimens based on treatment type and patient feedback. While improvements in local anaesthetic agents and delivery techniques have significantly reduced intra-operative discomfort, as evidenced by the low post-anaesthesia VAS scores across both groups, this study highlights that post-treatment pain remains a significant concern, particularly in endodontics. These findings highlight the importance of combining both pharmacological and non-pharmacological pain control measures, including patient education, psychological reassurance, and tailored follow-up care.

## Conclusion:

Endodontic treatment was associated with higher pain levels both during and after the procedure compared to periodontal therapy. The pain intensity was significantly higher in the endodontic group, particularly during the post-operative period. Data suggests that while both treatments caused some level of discomfort, endodontic procedures resulted in more prolonged and intense pain, thereby requiring more intensive pain management strategies.

## References

[1] Becker D.E (2010). Anesth Prog..

[2] Bhuva B (2025). Br Dent J..

[3] https://www.ncbi.nlm.nih.gov/books/NBK554590/.

[4] Sunitha V.R (2008). J Conserv Dent..

[5] Chen E (2009). Int J Dent..

[6] Nixdorf D.R (2015). J Endod..

[7] Baagil H (2023). Medicina (Kaunas)..

[8] Canakci C.F (2007). The Journal of the American Dental Association..

[9] Sirintawat N (2017). J Dent Anesth Pain Med..

[10] Maggirias J (2002). Community Dentistry and Oral Epidemiology..

